# Chromosome 19q13 disruption alters expressions of *CYP2A7*, *MIA* and *MIA-RAB4B* lncRNA and contributes to FAP-like phenotype in *APC* mutation-negative familial colorectal cancer patients

**DOI:** 10.1371/journal.pone.0173772

**Published:** 2017-03-17

**Authors:** Lai Fun Thean, Yu Hui Wong, Michelle Lo, Carol Loi, Min Hoe Chew, Choong Leong Tang, Peh Yean Cheah

**Affiliations:** 1 Department of Colorectal Surgery, Singapore General Hospital, Singapore; 2 Saw Swee Hock School of Public Health, National University of Singapore, Singapore; 3 Duke-NUS Medical School, National University of Singapore, Singapore; Heidelberg University, GERMANY

## Abstract

Familial adenomatous polyposis (FAP) is an autosomal-dominantly inherited form of colorectal cancer (CRC) caused by mutation in the adenomatous polyposis coli (*APC*) gene. Our ability to exhaustively screen for *APC* mutations identify microsatellite-stable and *APC*-mutation negative familial CRC patients, enabling us to search for novel genes. We performed genome-wide scan on two affected siblings of one family and 88 ethnicity- and gender-matched healthy controls to identify deletions shared by the siblings. Combined loss of heterozygosity, copy number and allelic-specific copy number analysis uncovered 5 shared deletions. Long-range polymerase chain reaction (PCR) confirmed chromosome 19q13 deletion, which was subsequently found in one other family. The 32 kb deleted region harbors the *CYP2A7* gene and was enriched with enhancer, repressor and insulator sites. The wildtype allele was lost in the polyps of the proband. Further, real-time RT-PCR assays showed that expressions of *MIA* and *MIA-RAB4B* located 35 kb upstream of the deletion, were up-regulated in the polyps compared to the matched mucosa of the proband. *MIA-RAB4B*, the read-through long non-coding RNA (lncRNA), *RAB4B*, *PIM2* and *TAOK1* share common binding site of a microRNA, miR-24, in their 3’UTRs. *PIM2* and *TAOK1*, two target oncogenes of miR-24, were co-ordinately up-regulated with *MIA-RAB4B* in the polyps, suggesting that *MIA-RAB4B* could function as competitive endogenous RNA to titrate miR-24 away from its other targets. The data suggest that the 19.13 deletion disrupted chromatin boundary, leading to altered expression of several genes and lncRNA, could contribute to colorectal cancer via novel genetic and epigenetic mechanisms.

## Introduction

Colorectal cancer (CRC) is one of the most frequent cancers and a leading cause of cancer mortality in the developed world. There are two main autosomal-dominantly inherited CRC, familial adenomatous polyposis (FAP) and hereditary non-polyposis colorectal cancer (HNPCC). The former is caused mainly by germline mutation in the adenomatous polyposis coli (*APC*) gene [1–3] and the latter by mismatch repair genes (MMR) characterized by microsatellite instability [4–6]. Our ability to exhaustively screen for these genes have enabled us to identify familial CRC patients who are microsatellite-stable and *APC*-mutation negative, implying that the underlying defect is in other genes although the clinical manifestation and mode of inheritance is similar to FAP [7, 8].

We have previously identified a copy number variant (CNV) region that regulates *PPM1L*, a novel serine-threonine phosphatase, in *APC*-mutation negative familial CRC patients with aggressive polyposis via genome-wide scan [9]. A recent comprehensive survey has also reported that microsatellite stable, non-polyposis familial Caucasian patients had increased CNV burden indicating further that structural variation could play significant role in familial colorectal tumorigenesis [10]. In this study, we embarked on the search of novel genes in one of the *APC*-mutation negative families with attenuated polyposis in which other possible tumour suppressors had been ruled out. Whole genome genotyping and CNV analysis identified a 32 kb genomic deletion at chromosome 19q13 shared by both affected siblings of the family that was not found in 88 ethnicity- (Chinese) and gender- (male) matched healthy controls.

We showed that the expression of *CYP2A7* located within the deletion was down- regulated. We further showed that *MIA*, and its run-through lncRNA, *MIA-RAB4B*, located 35 kb upstream of the deletion, were specifically up-regulated in the polyps compared to matched colonic mucosa of the proband but was correspondingly down regulated in that of *APC* mutation-positive FAP patients. Moreover, *MIA-RAB4B* could possibly function as a competitive endogenous RNA (ceRNA) to titrate away micro-RNA 24, resulting in the up-regulation of its other target oncogenes, *PIM2* and *TAOK1*.

The results imply that the 32 kb genomic deletion at chromosome 19q13 remove regulatory elements and reposition several upstream oncogenes, leading to their activation thus contributing to tumorigenesis.

## Materials and methods

### Patient samples and phenotypic classification

Polyposis patients were registered with the Singapore Polyposis Registry. The diagnostic criteria for attenuated FAP were the presence of 20 or more adenomas throughout the colon and rectum as documented in the patient’s histopathological records. All probands were diagnosed at colonoscopy or colectomy. The proband was interviewed and the detailed pedigree of the family and clinical history of proband and other affected members documented. Clinicopathological data were retrieved from histopathology reports and patient medical records of Singapore General Hospital (SGH). Peripheral lymphocyte and tissue specimens were collected with written informed consent. This study was approved by SingHealth Centralized Institutional Review Board B.

### Exclusion of *APC*, *MutYH*, *BMPR1A*, *PTEN*, *p53*, *POLD1*, *POLE* and *NTHL1* germline mutations

*APC* germline mutation was excluded by a combination of the protein truncation test, multiplex ligation-dependent probe amplification (MLPA) and differential expression assay as previously described [7]. *MutYH*, *BMPR1A*, *PTEN* and *p53* germline mutations were excluded by amplification of exonic fragments followed by Sangers’ sequencing on ABI 3100 automated sequencer. Exclusion of *POLD1*, *POLE* and *NTHL1* were performed on the mutation hot spots c.1421T>C, c.1433G>A (*POLD1*), c.1270C>G (*POLE*), and c.268C>T (*NTHL1*) respectively. The sequences of the primer pairs used are available upon request.

### Microsatellite instability assay

Microsatellite instability assay was performed on matched mucosa and tumour samples using the MSI Analysis System from Promega (Madison, WI). Briefly, 1-2ng DNA from matched mucosa and tumour tissues was used in each multiplex PCR reaction according to manufacturer’s protocol [11]. The fluorescence labeled PCR products were separated on ABI PRISM 310 Genetic Analyzer and analyzed using the ABI Gene Scan Analysis software. A microsatellite-stable tumour (MSS) is a tumour with identical profile as matched mucosa in all 5 mononucleotide markers.

### Genome-wide genotyping and copy number analysis

Genomic DNA was extracted using the high salt method [12]. Whole-genome scan was performed with Affymetrix GeneChip Human Mapping SNP Array 6.0 consisting 906, 600 SNPs and 946,000 copy number probes as per the manufacturer’s instructions (Affymetrix, Santa Clara, CA). Arrays were scanned with the Affymetrix 3000-7G scanner and analysed with the Genotyping Console v3. Only arrays with call rate 97% or more were accepted for further data processing [13].

The CEL (cell intensity) files from the genome-wide scan were imported into Partek Genomics Suite for bioinformatics analysis. The CEL files were subjected to principal component and copy number analysis. The copy number tool made use of the Hidden Markov Model (HMM). Unpaired copy number analysis were performed on CEL files from the lymphocytic DNAs of the affected siblings and that of the 88 ethnicity- and gender-matched controls as reference baseline to identify deletions common to both siblings but not found in the reference. Three different algorithms, loss of heterozygosity (LOH), copy number and allelic-specific copy number analysis were performed using default settings of the software. Only deletions that were called by all three algorithms were followed-up. In addition, the CEL files from the two polyp DNAs of the proband were compared to that of the matched lymphocytic DNA as reference (paired copy number analysis) to determine whether the second allele was deleted.

### Data mining the confirmed deleted region

The flanking region surrounding the genomic deletion was imported into the Repeat Masker program (http://www.repeatmasker.org/) to search for repetitive sequences. The 1Mb region encompassing the deletion was further interrogated with the NCBI (http://www.ncbi.nlm.nih.bov/), Ensembl (http://www.ensembl.org/) and the UCSC (http://genome.ucsc.edu/) databases with emphasis on the chromatin modification tracks under ENCODE to identify regulatory elements.

### Long range polymerase chain reaction

The reactions were performed with the Expand long Range, dNTPack (Roche). The 25-μl reaction volume contained 1X expand long range buffer with 12.5mM MgCl_2_, 500μM PCR nucleotide mix, 0.3μM of each primer, 1% DMSO, 3.5U enzyme mix and 250 ng template DNA. The internal control is a 6kb *BMPR1A* amplicon at chromosome 10q23. The amplification reaction consisted of denaturing at 92°C for 2 min, followed by 10 cycles of 92°C for 10s, 63°C for 15s and 68°C for 8 min, 25 cycles of 92°C for 10s, 63°C for 15s and 68°C for 8 min plus an extra 20s elongation per cycle, and final elongation at 68°C for 7 min as previously performed [14].

### Real-time RT-PCR assay and primers

cDNA was synthesized from total RNA using ABI High-Capacity cDNA Reverse Transcription kit according to the protocols of the manufacturer (http://www3.appliedbiosystems.com/cms/groups/mcb_support/documents/generaldocuments/cms_042557.pdf). The cDNA generated was used as template in real-time PCR reactions with ABI SYBR-Green PCR master mix on MicroAmp^®^ Optical 384-well reaction plate and ran on ABI 7900HT. All reactions were performed in quadruplicate. The primers for the amplifications were available upon request. Validation experiments verified that the efficiencies of amplification of the target genes and beta-actin, the internal reference, were approximately equal. Hence, relative quantification for each gene (2^-ΔΔCt^) in the polyps compared to the matched mucosa was determined using the comparative Ct method [15].

## Results

### Identification of *APC*-mutation negative familial CRC patients

**Fig 1:**
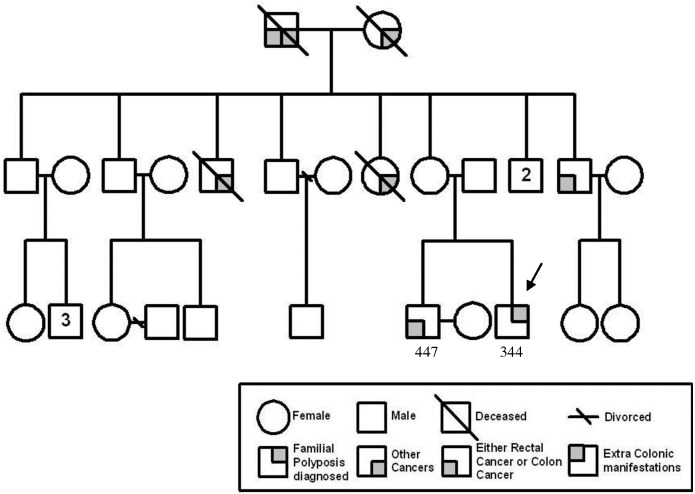
Pedigree of *APC* mutation-negative familial CRC family 1. Arrow indicates the proband.

We screened for *APC* germline mutation in FAP patients by a combination of techniques that enabled us to achieve a high *APC* mutation detection rate of over 90% in the families registered in the Singapore Polyposis Registry [7]. Consequently, we were able to identify a sub-class of familial CRC patients clinically diagnosed as FAP or attenuated-FAP but were *APC*-mutation negative. One of these families is represented in Fig 1. The proband, 344, was diagnosed as an attenuated FAP patient with 25 adenomatous polyps, precursors to carcinomas, at age 23. Subsequently, the elder brother, 447, presented with multiple mixed (adenomatous as well as juvenile and hyperplastic) polyps and cecal mucinous adenocarcinoma at age 35 and underwent right hemicolectomy. No FAP-associated extracolonic manifestation was recorded.

Microsatellite instability assay performed indicated that the tumour is microsatellite- stable. We considered the possibility that mutation in other tumour suppressors could have caused the clinical phenotype. We searched for germline mutation in *MutYH*, *BMPR1A*, *PTEN*, *p53*, *POLD1*, *POLE and NTHL1* genes by direct exonic Sanger’s sequencing and found the proband to be mutation-negative in these genes. It is thus likely that the disease-causing gene lies elsewhere although the clinical phenotype mimics that of FAP patients.

### Prediction of 5 deletions shared by the two affected siblings

We embarked on genome wide scan of the lymphocytic and polyp DNA of the two affected siblings, 344 and 447, in this family (Fig 1) and 88 ethnicity- and gender-matched healthy controls using the Affymetrix SNP 6 arrays.

We imported the CEL files into third-party software, Partek Genomics Suite, and searched for germline deletions that were shared by these two brothers but were not found in the 88 healthy controls. The deletions were called only if they were identified by all three algorithms applied, loss of heterozygosity, copy number and allelic-specific copy number analysis. Partek Genomics Suite predicted 5 genomic deletions in four different chromosomes (Table 1).

**Table 1:** Five predicted germline deletions shared by the two affected siblings.

Chr	Start*	Stop*	No. of Markers	Total size (bp)	Associated genes
1q32.2	210076338	210085268	3	8931	None
3q28	189362217	189367813	11	5597	*TP63*
11q11	55376390	55453292	55	76903	*OR4P4*, *OR4S2*, *OR4C6*, *OR4V1P*, *OR4P1P*
19q13.2	41360272	41392154	32	31883	*CYP2A7*
19q13.31	43700184	43761448	23	61265	*PSG4*, *CEACAMP10*, *LOC284344*, *PSG9*

* Physical positions based on GRCh37/hg19

### Validation of the genomic deletion at chromosome 19q13

We ranked the 5 predicted deleted regions by the candidate genes residing within the deletions (Table 1). Other than the two highest ranked chromosomes 3q28 and 19q13.2 regions, the other three regions harbor either no candidate gene (chromosome 1q32.2) or genes unlikely to be implicated in polyposis or cancer such as olfactory receptor (chromosome 11q11) or pregnancy-specific genes (chromosome 19q13.31). We decided to validate the first two highest ranked regions at chromosomes 3q28 and 19q13 with a predicted deletion size of approximately 6 kb and 32 kb respectively by a secondary long-range PCR assay. Primers approximately 3 kb from the predicted deletion sites at 3q28 amplified a 10 kb TP63 fragment in the lymphocytic DNAs of 273 (the ethnicity- and gender-matched healthy control), 344 and 447 (affected siblings) as well as in two different polyp DNAs of 344 with approximately equal intensity after normalization to the internal control *BMPR1A* (S1 Fig). Hence, the predicted 6 kb deletion at 3q28 (encompassing *TP63*) was not validated.

**Fig 2:**
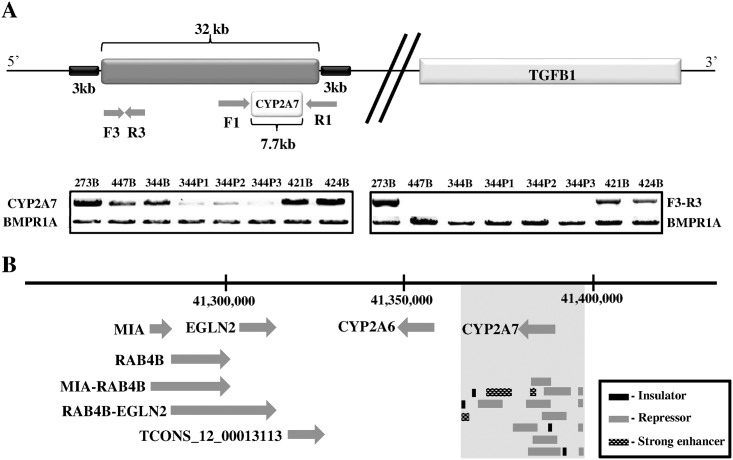
**(A) Schematic of the 32 kb genomic deletion at chromosome 19q13.** The gel pictures represent the long range PCR products amplified with primer pairs F1-R1 and F3-R3. The internal control is a 6 kb *BMPR1A* fragment from chromosome 10q23. 273, male healthy control; 344, proband; 447, another affected sibling; 421 and 424; *APC* mutation-positive FAP patients; B, blood; P, polyp. **(B) The genes and lncRNAs in the 35 kb region upstream of the deletion.** The deletion is shown as vertical grey block, with relevant regulatory element tracks extracted from the UCSC browser.

We then interrogated the predicted deleted region at 19q13 with a pair of long-range PCR primers (F1 and R1) which amplified a 7.7 kb fragment encompassing the *CYP2A7* gene (Fig 2A). The amplified fragments from the lymphocytic DNA of the two affected siblings, 344 and 447, were only about half the intensity of 273, 421 and 424 after normalization to the internal control, indicating that the template DNA was probably halved. 421 and 424 were the *APC* mutation-positive controls. The fragment intensity was further reduced in the DNA isolated from the three polyps of 344 suggesting that the deletion could now be on both chromosomes. Copy number plot generated from the paired analysis between 344 and the polyps P1 and P2 showed that indeed a similar region of 28.5 kb is deleted in the polyps (S2 Fig, S1 Table). We designed another set of PCR primers (F3-R3) spanning the intergenic region within the deletion and showed further that the expected 2.5 kb fragment was clearly amplified in 273, 421 and 424 only (Fig 2A).

We screened the probands of another eleven *APC* mutation-negative families using the same set of primers (F1-R1) and found this deletion in one other Chinese family.

### *In silico* analysis of the 1 Mb region encompassing the deletion

Interrogating flanking regions of the deletion with the repeat masker program revealed multiple identical repetitive sequences of the MIR and Alu families (S3 Fig). Since the similarity scores of the Alu families of elements flanking the breakpoints are higher than the MIR’s, the genomic deletion is likely to be mediated by a homologous recombination event involving the Alu elements, as previously reported in a Singapore FAP family [16].

It is interesting to note that the only gene within the genomic deletion at 19q13 is *CYP2A7*, a member of a nicotine-metabolizing family of enzymes that was not previously considered as a typical tumour suppressor [17]. Hence, we speculated that the 32 kb deletion may harbour regulatory elements that could enhance or repress neighbouring genes as previously reported [9]. *In silico* analysis of the deleted region with the UCSC Browser tracts revealed a chromatin three-dimensional architecture highly inundated with many enhancer, insulator and repressor sites (Fig 2b). Insulators divide the chromosome into topologically associating domains (TAD) marked by the CTCF proteins [18]. Hence, the deletion could remove a TAD and alter the regulation of candidate tumour suppressors or oncogenes in the vicinity [19, 20].

We scanned a 1 Mb region encompassing the genomic deletion and identified several candidate genes. *TGFβ1*, located about 400kb downstream of the deletion (Fig 2A), is a ligand in the TGFβ signalling pathway that is known to play important role in colorectal tumourigenesis [21]. *MIA*, *RAB4B* and *EGLN2* are three genes located in close proximity and about 35 kb upstream of the deletion (Fig 2B). *MIA* and *EGLN2* were previously implicated in tumourigenic processes such as cellular proliferation and invasion [22, 23]. *RAB4B* is a small GTPase in the RAS signalling pathway, the dysregulation of which led to cancers in many organs [24]. Two related read-through transcripts that were predicted to be long non-coding RNA (lnc-RNA), *MIA-RAB4B* and *RAB4B-EGLN2*, interspersed these three genes. In addition, another lnc-RNA in the vicinity, lncRNA_TCONS_|2_00013113, was predicted by the Encode “transcript of uncertain coding potential (TUCP)” algorithm (Fig 2B).

### Expression of *CYP2A7* and *TGFβ1*

Several lines of evidence have indicated that disruption of chromatin boundaries by deletion, leading to heterochromatin spreading and enhancer or silencer blocking, are stage- and tissue-specific [18, 25]. We therefore investigated the expressions of these candidate genes in the polyps compared to that of the matched mucosa in affected sibling 344. This was then contrasted to that of 421, an *APC* mutation-positive FAP patient with no deletion at 19q13.

**Fig 3:**
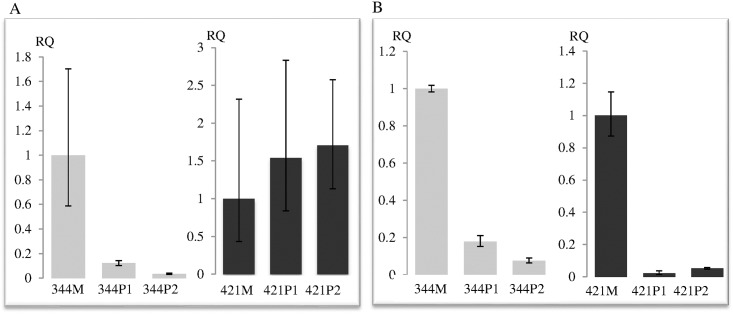
**Relative Quantitation (RQ) of (A) *CYP2A7* and (B) *TGFβ1* transcripts in the polyps normalized to matched mucosa of 344 and 421 respectively**. Vertical bar denotes maximum and minimum RQ of each specimen. M, mucosa; P, polyp.

We interrogated first the expression of *CYP2A7* and *TGFβ1* by real-time RT-PCR assay. The expression of *CYP2A7* was diminished ten-fold or more in the two polyps of 344 compared to the matched mucosa (Fig 3A). On the contrary, the expressions of *CYP2A7* in the polyps of 421 were slightly elevated compared to that of the matched mucosa. The expression of *TGFβ1*, however, was similarly down-regulated in the polyps of both 344 and 421 (Fig 3B).

### Expression of *MIA*, lncRNAs and *EGLN2*

We investigated next the expressions of the candidate genes 5’ to the genomic deletion, *MIA*, *EGLN2*, and their related read-through lncRNAs, *MIA-RAB4B* and *RAB4B-EGLN2*. It was not possible to interrogate the expression of *RAB4B* as it was not possible to design primers unique to this transcript alone.

**Fig 4:**
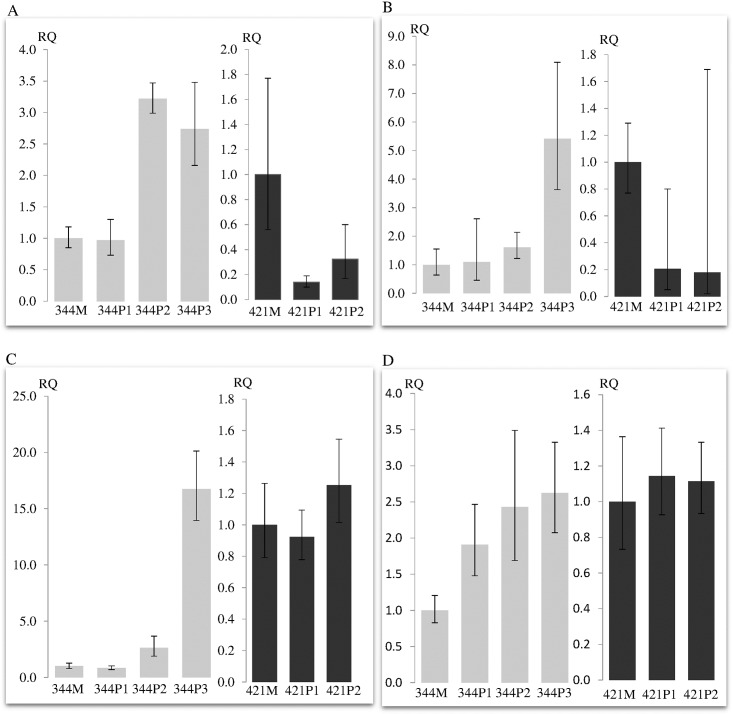
**Relative quantitation of (A) *MIA* (B) *MIA-RAB4B* (C) *PIM2* and (D) *TAOK1* transcripts in the polyps normalized to matched mucosa of 344 and 421 respectively.** *MIA-RAB4B* is the read-through lncRNAs of *MIA* and *RAB4B*. *PIM2* and *TAOK1* are two other target oncogenes of miR-24.

Although the expressions of *MIA* and *MIA-RAB4B* were consistently down-regulated in the polyps of 421 compared to the matched mucosa, the expressions of these genes were one to five fold up-regulated in the polyps of 344 (Fig 4A and 4B). The minimum and maximum relative quantitation (RQ-min and RQ-max) for *MIA-RAB4B* was large in the polyps of 421 as the expression of this read-through lncRNA is very low and thus cannot be accurately measured. The expression of *EGLN2* and the second read-through lncRNA, *RAB4B-EGLN2*, remain in approximately the same level in the polyps of both 344 and 421 compared to their respective mucosa. Furthermore, the expression of another lncRNA in close proximity, lncRNA_TCONS_|2_00013113 (Fig 2B) remained at barely detectable level in the polyps of both 344 and 421 (data not shown).

### Expression of a subset of miR-24 regulated genes

*In silico* analysis with NCBI database indicated that *MIA-RAB4B* and *RAB4B* share the same 3’ untranslated region (UTR) including the seed recognition sites for miR-24. This suggests that *RAB4B* expression could be affected by the up-regualtion of *MIA-RAB4B* via miRNA titration [26]. The expression of *RAB4B*, however, cannot be investigated independently of *MIA-RAB4B* and *RAB4B-EGLN2* (Fig 2B). We therefore searched for other miR-24-regulated, putative *MIA-RAB4B* ceRNA targets. Interrogation using miRNA target prediction softwares identified *PIM2*, *TAOK1*, and *MAPK7* as other oncogeneic targets of miR-24. These genes were identified by all three predictive softwares, TargetScan, MiRanda and PicTar.

Interestingly, the expressions of *PIM2* and *TAOK1* were increased up to fifteen and two and half folds respectively in the polyps of 344 compared to the matched mucosa whilst the expressions of these genes in the polyps of 421 remained constant (Fig 4C and 4D). There was, however, no difference in the expression of *MAPK7* in the polyps of 344 and 421 compared to their matched mucosa (data not shown).

## Discussion

In this study, we report the identification of a 32 kb chromosome 19q13 deletion that specifically disrupts the expressions of *CYP2A7*, *MIA* and *MIA-RAB4B* lncRNA in an *APC* mutation-negative patient compared to two different *APC* mutation-positive patients. The overall workflow of this study is summarized in Supporting S4 Fig. It is postulated that this germline deletion could mimic the loss of *APC* and contribute to the initiation of the FAP-like phenotype in this *APC* mutation-negative microsatellite stable family 1.

Germline mutations in other known polyposis and cancer causing tumor suppressors such as *MUTYH*, *BMPR1A*, *PTEN*, *TP53*, *POLD1*, *POLE* and *NTHL1* were also excluded. The polyps in proband 344 and affected sibling 447 of family 1 are adenomas with just a few mixed polyps documented in 447; hence it is unlikely that the *SMAD4* gene (responsible for juvenile polyposis) is disease-causing. Chromosome 15q13.3 (harbouring *GREM1*) region was not amplified in both affected siblings 344 and 447 compared to the 88 ethnicity- and gender- matched controls (Table 1). Recent studies have not found *GALNT12* at chromosome 9q22-33 to be a highly penetrant gene in Mendelian syndromes [27, 28]. We have also not identified this locus as a shared deleted region in family 1 which has autosomal dominant inheritance (Table 1).

Copy number analysis by three different algorithms of the dataset created from genome-wide scan of the two affected siblings of family 1 (Fig 1) and 88 ethnicity- and gender-matched healthy controls revealed 5 shared deletions in the siblings (Table 1). The deletion at chromosome 19q13 (but not the deletion at 3q28) was validated by long-range PCR (Fig 2A, S1 Fig). The data reemphasize that bioinformatics analysis has to be corroborated by independent secondary assay.

Notably, the 32 kb deletion encompassed the *CYP2A7* gene only (Fig 2B). Both copies of *CYP2A7* gene appeared to be lost in the polyps of the proband, 344, by bioinformatics analysis as well as long-range PCR experiments (Fig 2A and S2 Fig). The loss of the wildtype allele is consistent with this deletion functioning as a tumor suppressor. Thus, it was not surprising that *CYP2A7* expression in the polyps compared to the matched mucosa of 344 was very much down-regulated while the expression in the polyps of 421 (the *APC* mutation-positive patient with no deletion at 19q13) were similar to the matched mucosa (Fig 3A).

No miRNA gene resides in this 32 kb region. However, the region is inundated with several strong enhancers, repressors and insulators (Fig 2B). Thus, the deletion is predicted to disrupt chromatin architecture and consequently alter the regulation of neighbouring genes [19, 20]. We showed that this cis-regulation was specific as only the expressions of *MIA* and *MIARAB4B*, located 35 kb upstream of the deletion, were up-regulated in the polyps of 344 but consistently down-regulated in the polyps of 421 (Fig 4A and 4B). The expression of *TGFβ1*, on the other hand, was similarly down-regulated in the polyps of both 344 and 421, suggesting that the deletion did not affect *TGFβ1* regulation in the colonic mucosa and that regulating element(s) for *TGFβ1* expression resides outside of the deletion and was not affected by the chromatin repositioning (Fig 3B).

Melanoma inhibitory activity (*MIA*) has been shown to increase the invasiveness of cancer cells not only in melanomas but also in solid tumors such as pancreatic and gastric carcinomas [29]. Hence, *MIA* up regulation in the polyps of 344 is likely to lead to increased invasiveness in CRC (Fig 4A). Notably, the read-through lncRNA, *MIA-RAB4B*, was also up-regulated (Fig 4B). A recent study has proposed a novel mechanism whereby the high mobility group AT-hook 2 (HMG2A) transcript promotes non-small cell lung cancer progression by competing with transforming growth factor β receptor III (*Tgfbr3*) for let-7 binding [26]. Since *MIA-RAB4B* and *RAB4B* share the same seed recognition site of miR-24, we wondered whether a similar mechanism could operate in this instance i.e. increased expression of *MIA-RAB4B* could compete away the binding of miR-24 to *RAB4B*, thus reducing its degradation. This derepression could conceivably be extended to other targets of miR-24. We investigated the expressions of other target oncogenes of miR-24 as the expression of *RAB4B* cannot be studied directly (since no *RAB4B* unique primers can be designed). The expressions of *PIM2* and *TAOK1* were up-regulated in the polyps of 344 but not in 421 (Fig 4C and 4D). The expression of *MAPK7* in the polyps of both 344 and 421, however, were similarly down-regulated compared to their matched mucosa. Thus, in contrast to *MAPK7*, the co-ordinated overexpression of *MIA-RAB4B*, *PIM2* and *TAOK1* suggests that the latter two oncogenes could be critical targets of *MIA-RAB4B* ceRNA. PIM2 is a serine-threonine kinase that was found to be overexpressed in various solid tumours and involved in anti-apoptotic, metabolic and cell cycle regulation [30–32]. TAOK1 is a member of sterile 20 (STE20)-like kinases previously implicated in microtubule organisation and play active role in cytoskeletal stability and mitotic progression [33, 34]. The overexpression of *PIM2* and *TAOK1* oncogenes in 344 but not 421 polyps implies that the dysregulation of these genes and consequently their participatory pathways could possibly mimic the dysregulation due to loss of *APC*.

The 32 kb genomic deletion at chromosome 19q13 disrupts chromatin organization and alters the expressions of several genes in two out of twelve (16.7%) of the *APC* mutation-negative familial CRC families screened. In healthy individuals, the frequency of copy number loss in this region from the combined Database of Genomic Variants (DGV) (http://dgv.tcag.ca/dgv/app/home) entries is 1.68% (n = 7620 individuals). Thus, this copy number loss is significantly (Fisher’s Exact Test, p = 0.017) enriched in a subset of familial CRC patients previously screened to be mutation-negative in other possible disease-causing genes such as *APC and BMPR1A*.

The main limitation of the study is the lack of samples from other members of family 1 to show co-segregation of the disease with the 32 kb genomic deletion. Furthermore, no polyps were available from the second affected sibling 447 for the expression studies. Nevertheless, the expressions of the various genes studied in the polyps of another *APC* mutation-positive patient, 424, closely resembles that of 421 (S5 Fig), indicating that the expressions of these genes were consistent for patients with different *APC* germline mutations and distinct from that of 344 polyps. Patient 421 has a novel *APC* codon 1309 deletion while patient 424 has whole *APC* gene deletion. Furthermore, there was a second somatic deletion in the wildtype allele consistent with this locus being important for polyp development. Thus, although it is conceivable that the causative gene may still remain to be discovered, the 32 kb genomic deletion could contribute to the FAP-like phenotype in these *APC* mutation-negative patients via both genetic and epigenetic mechanisms.

## Supporting information

S1 Fig The 10 kb TP63 product from long range PCR. The internal control is a 6 kb *BMPR1A* fragment from chromosome 10q23. 273, male healthy control; 344, proband; 447, another affected sibling; 421, *APC* mutation-positive FAP patient; B, blood; P, polyp.
(TIF)

S2 Fig Copy number plot from paired analysis between lymphocytic DNA of proband 344 and his polyp DNA P1 and P2. (TIF)

S3 Fig Schematic map of the 32 kb deletion and surrounding regions. Upper panel depicts the repetitive elements flanking the 32 kb deletion. Lower panel shows the homology of the 13.6 kb regions 5’ and 3’ to the deletion. The red region [880bp] is 98% identical; green a [1890bp] and green b [3398bp] regions are 97% and 98% identical; yellow region [7427bp] is 95% identical and grey region [1153bp] is 98% identical to each other.
(TIF)

S4 Fig Flow chart summarizing the overall results for *APC* mutation-negative familial CRC family with germline chr19q13 deletion. (TIF)

S5 Fig Relative Quantitation (RQ) of *MIA*, *MIA-RAB4B*, *PIM2* and *TAOK1* transcripts (Y-axis) in the polyps of 344 compared to that of 421 and 424, another *APC* mutation-positive patient (X-axis). Expressions of the transcripts are similar for both 421 and 424 but distinct from that of 344. Vertical bar denotes maximum and minimum RQ of each specimen. M, mucosa; P, polyp.
(TIF)

S1 Table Paired CN analysis between 344 and polyps by HMM segmentation workflow. (DOCX)
