# Supplementary figures and images for: Chromosome 19q13 disruption alters expressions of CYP2A7, MIA and MIA-RAB4B lncRNA and contributes to FAP-like phenotype in APC mutation-negative familial colorectal cancer patients
Source: PLoS One. 2017 Mar 17;12(3):e0173772. doi: 10.1371/journal.pone.0173772 (PMC5357012; doi:10.1371/journal.pone.0173772)

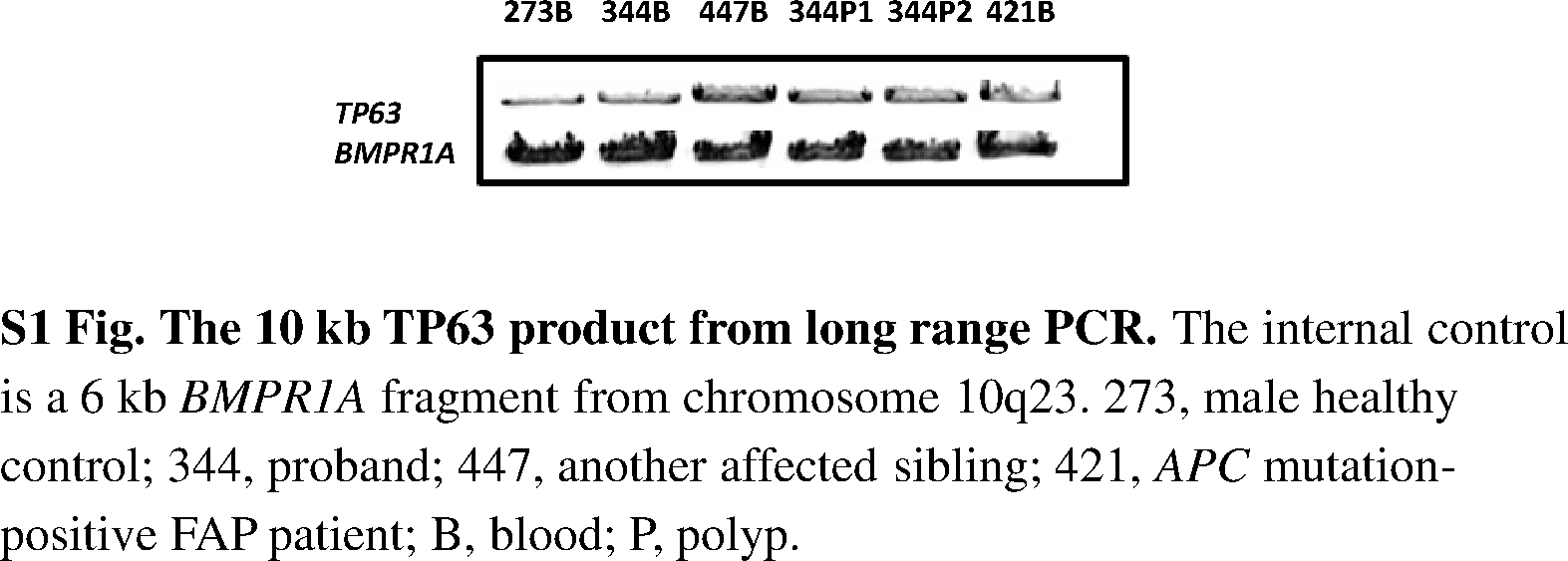

Supplement: S1 Fig — The internal control is a 6 kb BMPR1A fragment from chromosome 10q23. 273, male healthy control; 344, proband; 447, another affected sibling; 421, APC mutation-positive FAP patient; B, blood; P, polyp. (TIF) [file pone.0173772.s001.tif]

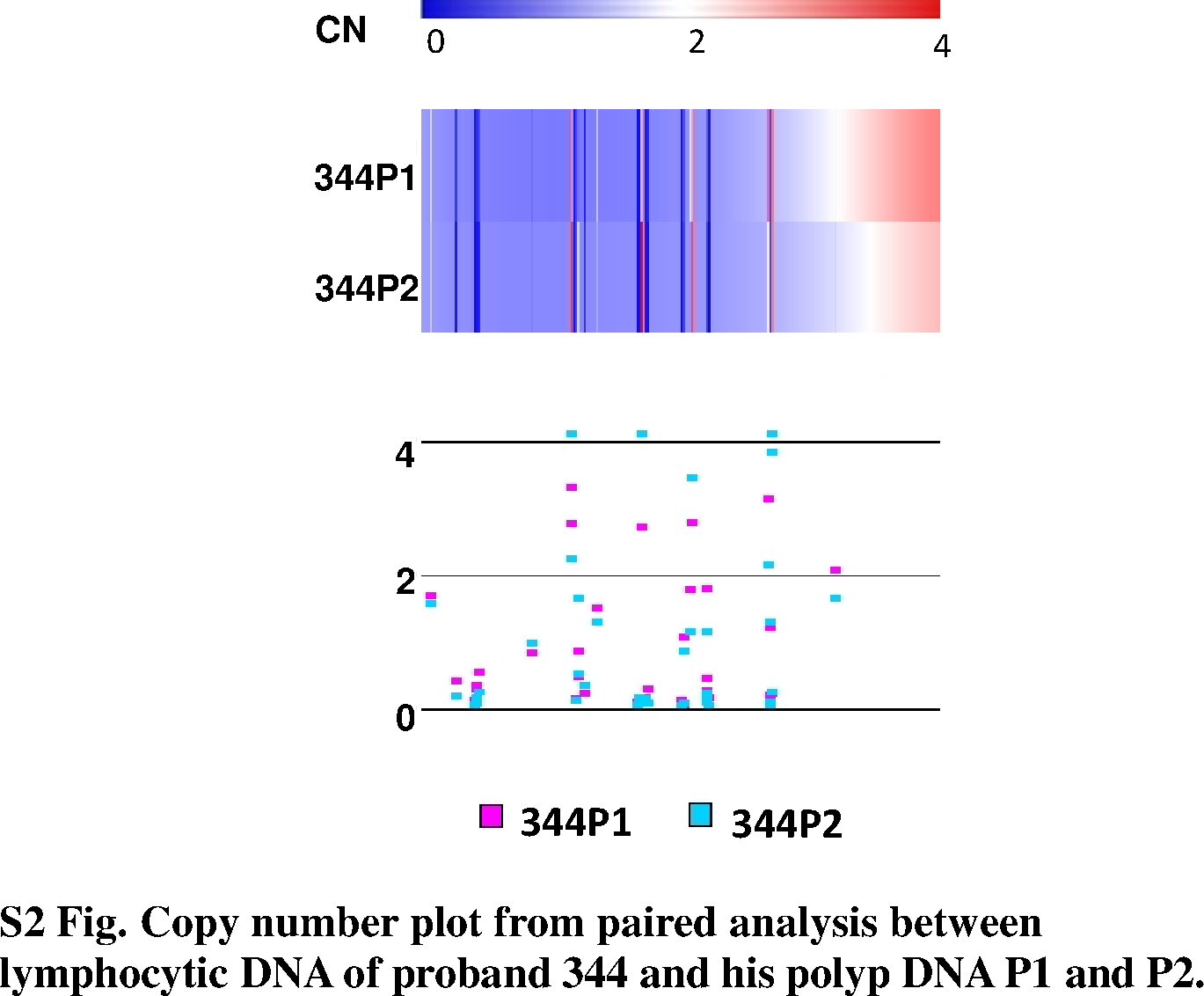

Supplement: S2 Fig — (TIF) [file pone.0173772.s002.tif]

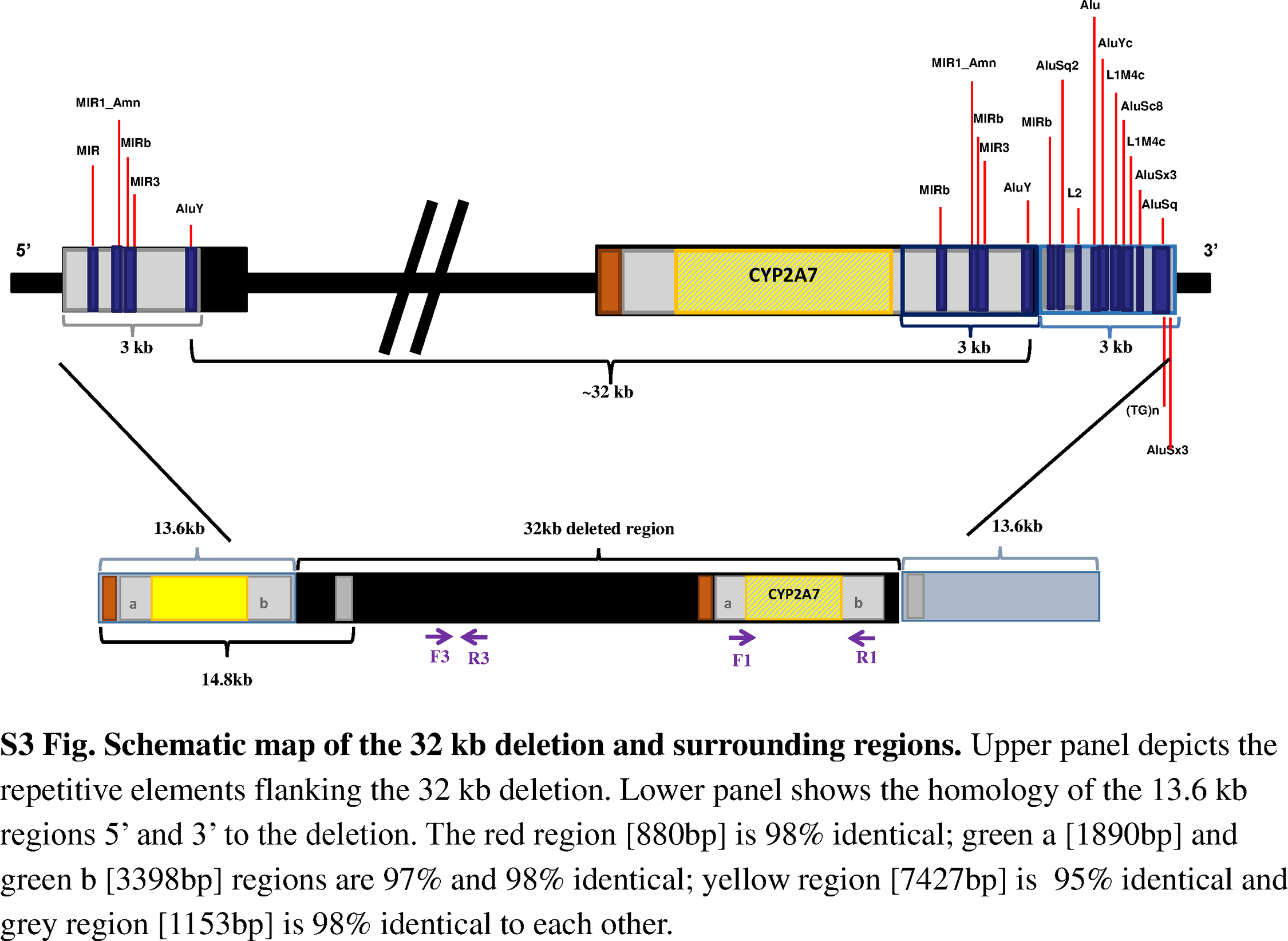

Supplement: S3 Fig — Upper panel depicts the repetitive elements flanking the 32 kb deletion. Lower panel shows the homology of the 13.6 kb regions 5’ and 3’ to the deletion. The red region [880bp] is 98% identical; green a [1890bp] and green b [3398bp] regions are 97% and 98% identical; yellow region [7427bp] is 95% identical and grey region [1153bp] is 98% identical to each other. (TIF) [file pone.0173772.s003.tif]

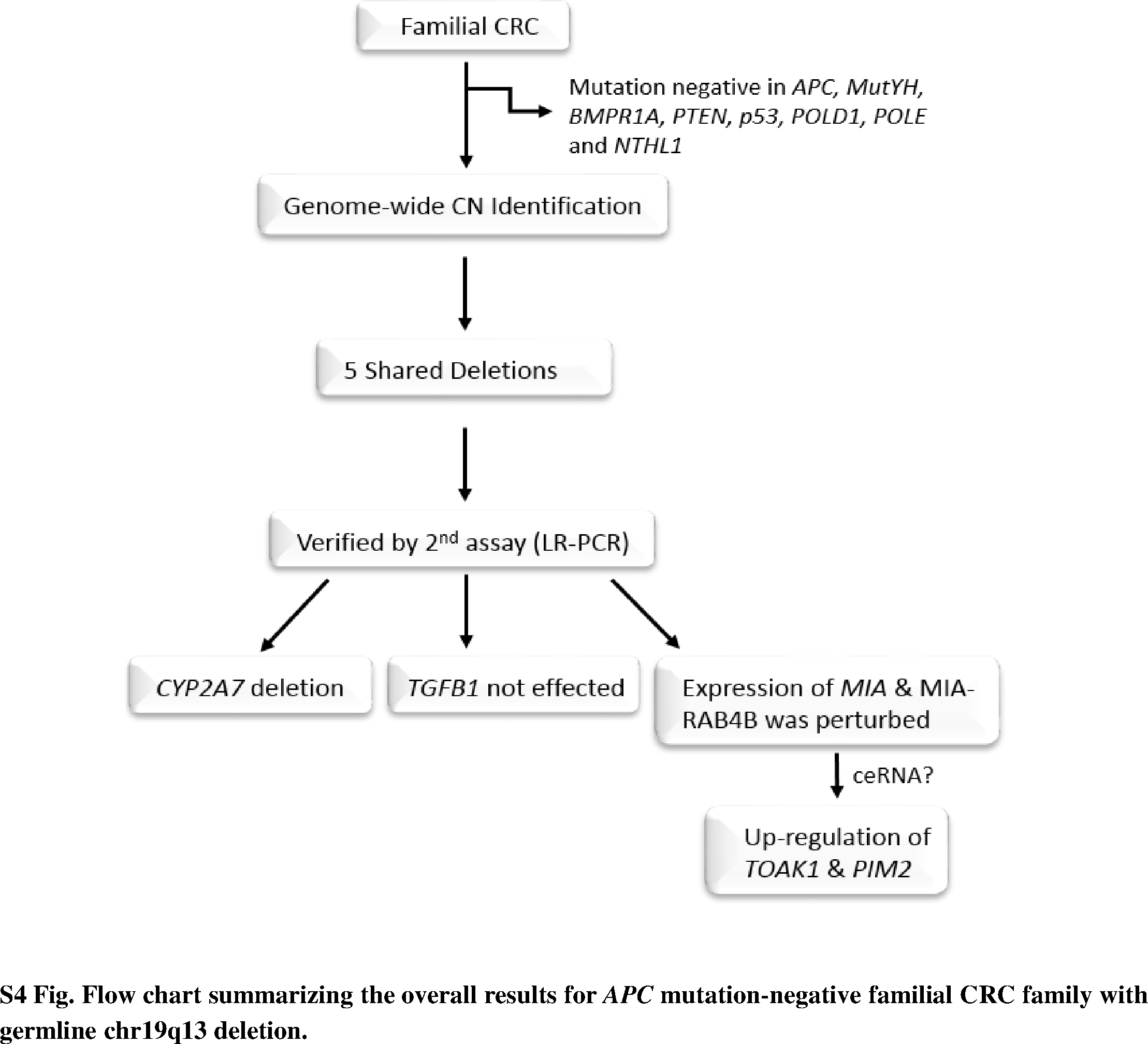

Supplement: S4 Fig — (TIF) [file pone.0173772.s004.tif]

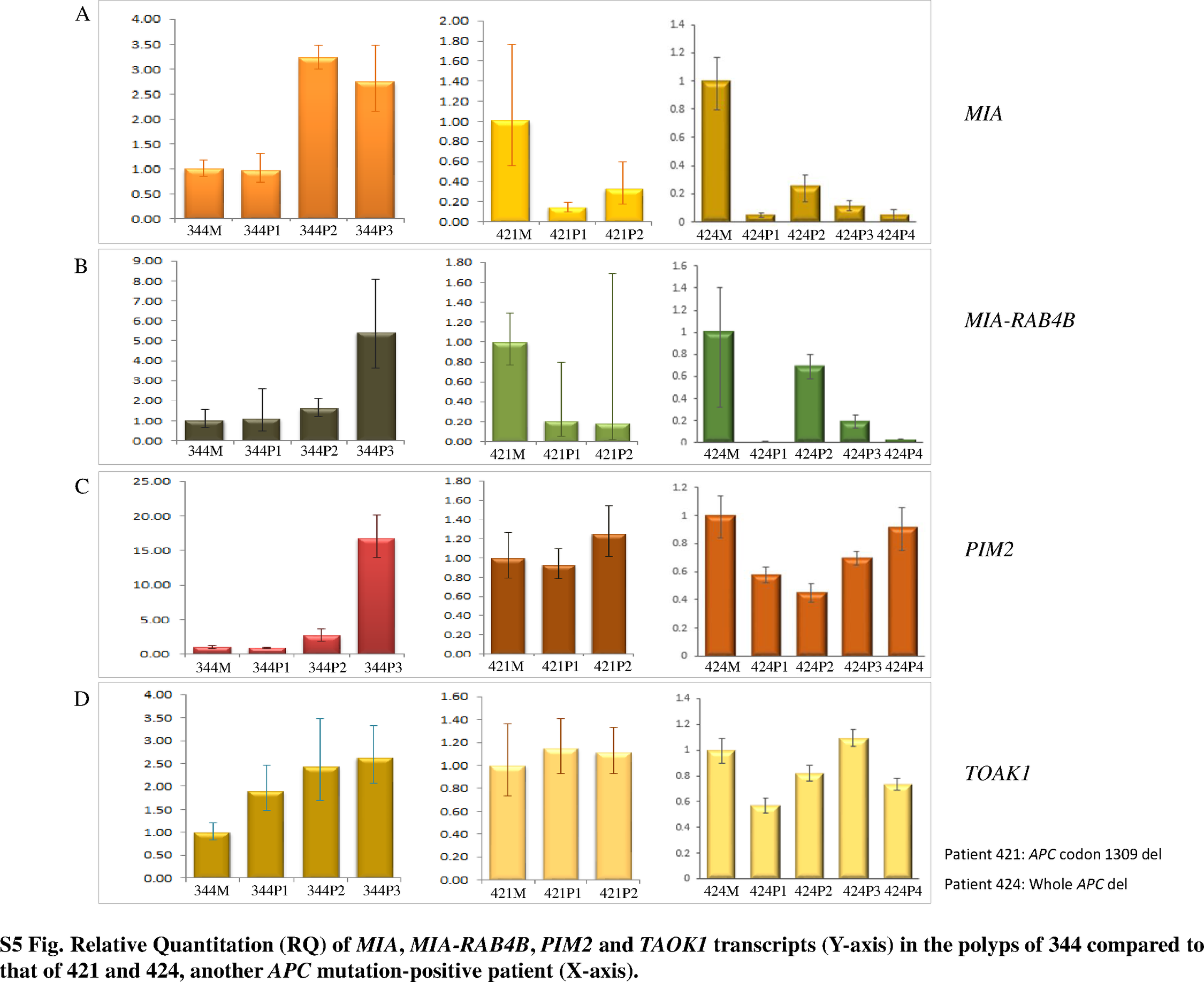

Supplement: S5 Fig — Expressions of the transcripts are similar for both 421 and 424 but distinct from that of 344. Vertical bar denotes maximum and minimum RQ of each specimen. M, mucosa; P, polyp. (TIF) [file pone.0173772.s005.tif]
