# Supplementary material for: Chromosome 19q13 disruption alters expressions of CYP2A7, MIA and MIA-RAB4B lncRNA and contributes to FAP-like phenotype in APC mutation-negative familial colorectal cancer patients
Source: PLoS One. 2017 Mar 17;12(3):e0173772. doi: 10.1371/journal.pone.0173772 (PMC5357012; doi:10.1371/journal.pone.0173772)
Supplement: S1 Table — (DOCX) [file pone.0173772.s006.docx]

**S1 Table.** Paired CN analysis between 344 and polyps by HMM segmentation workflow

| **Chr** | **Start** | **Stop** | **Total Amp** | **Amp av CN** | **AMP samples** | **Total Del** | **Del av CN** | **Del samples** | **# markers** | **bp** | **P1 mean** | **P1 CN status** | **P2 mean** | **P2 CN status** |
| --- | --- | --- | --- | --- | --- | --- | --- | --- | --- | --- | --- | --- | --- | --- |
| 19 | 46041772 | 46046708 | 0 | ? |  | 2 | 0.400743 | P1 P2 | 5 | 4937 | 0.648176 | del | 0.153309 | del |
| 19 | 46046708 | 46055540 | 0 | ? |  | 2 | 0.797935 | P1 P2 | 8 | 8833 | 0.648176 | del | 0.947693 | del |
| 19 | 46055540 | 46057378 | 0 | ? |  | 2 | 0.103248 | P1 P2 | 4 | 1839 | 0.10904 | del | 0.097456 | del |
| 19 | 46057609 | 46061042 | 0 | ? |  | 2 | 0.381148 | P1 P2 | 5 | 3434 | 0.608014 | del | 0.154283 | del |
| 19 | 46061042 | 46070322 | 0 | ? |  | 2 | 0.606644 | P1 P2 | 14 | 9281 | 0.608014 | del | 0.605274 | del |
| 19 | 46070322 | 46078390 | 1 | 2.80058 | P1 | 1 | 0.605274 | P2 | 1 | 8069 | 2.80058 | ampl | 0.605274 | del |
